# Comparison of deep learning-based segmentation and registration using pre-treatment contours for online rectal delineation in magnetic resonance-guided radiotherapy

**DOI:** 10.1016/j.phro.2025.100854

**Published:** 2025-10-21

**Authors:** Iris D. Kolenbrander, Koen M. Kuijer, Mark H.F. Savenije, Gert J. Meijer, Martijn P.W. Intven, Josien P.W. Pluim, Matteo Maspero

**Affiliations:** a IMAG/e Group, Department of Biomedical Engineering, Eindhoven University of Technology, Eindhoven, The Netherlands; b Eindhoven Artificial Intelligence Systems Institute, Eindhoven University of Technology, Eindhoven, The Netherlands; c Department of Radiotherapy, University Medical Center Utrecht, Utrecht, The Netherlands; d Computational Imaging Group for MR Diagnostics and Therapy, University Medical Center Utrecht, Utrecht, The Netherlands

**Keywords:** Online adaptive radiotherapy, Target contouring, Segmentation, Registration, Deep learning, Convolutional neural networks

## Abstract

**Background and Purpose:**

Deep learning promises accurate target contouring for online adaptive MR-guided radiotherapy (MRgRT) in rectal cancer. However, delineating the mesorectal clinical target volume (CTV) remains challenging. Integrating planning-based contours, delineated offline before treatment, can provide anatomical shape and boundary information. This study evaluated deep learning-based segmentation and registration models to determine the optimal approach for incorporating planning contours into online rectal contouring.

**Materials and Methods:**

Deep learning-based segmentation and registration models, both U-Nets, were developed using MRI of 104 rectal cancer patients, split into 68, 14, and 22 training, validation, and testing subjects. The segmentation model used the planning CTV and daily fraction MRI, while the registration model used the planning MRI and CTV and the daily fraction MRI. The models were compared in terms of contour accuracy (maximum Hausdorff distance (HD), Dice, and a qualitative score) and robustness against domain shifts.

**Results:**

When incorporating the planning contour, the segmentation and registration models achieved comparable median HD values of 9.3 mm (interquartile range, IQR: 7.1-12.1) and 10.2 (8.2-12.4) (p=0.18), respectively. However, segmentation achieved lower HD values in the middle and cranial regions of the target (HD_middle_: 5.3 mm (4.3-6.6) vs. 6.0 mm (4.8-8.0), p<0.05; HD_cranial_: 7.6 mm (6.3-10.7) vs. 9.6 mm (7.5-11.9), p<0.05). In addition, segmentation resulted in more clinically acceptable contours (9/10 versus 3/10) and was more robust to rectum volume variations than registration.

**Conclusion:**

Deep learning-based segmentation was identified as the optimal approach for incorporating the planning CTV into online rectal delineation in MRgRT.

## Highlights

• Using pre-treatment contours improved daily target contour accuracy by 15%–46%.
• Segmentation vs registration: contour accuracy and robustness to domain shifts.
• Segmentation outperformed registration by 11%–20% in mid and cranial regions.
• Rectal volume changes can reduce segmentation and registration contour accuracy.

## Graphical abstract

**Figure:**
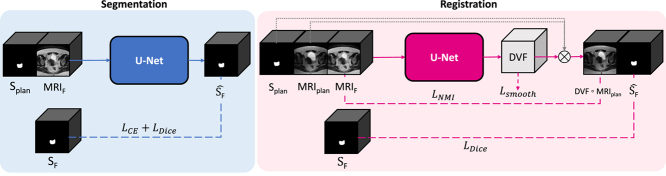

## 1 Introduction

Accurate delineation of the mesorectal clinical target volume (CTV) and surrounding organs-at-risk (OARs) is crucial for effective online adaptive magnetic resonance imaging (MRI)-guided radiotherapy (MRgRT). Radiation oncologists use deformable registration to propagate contours from a reference MRI to the daily scan. However, the produced contours often require further manual adjustments [1], which can take over ten minutes [1], [2], during which intra-fractional anatomical changes, such as organ filling and peristalsis, may occur [3]. These changes can compromise target coverage and increase radiation exposure to OARs. Therefore, more efficient and accurate contouring methods are essential to improving MRgRT delivery.

Deep learning has demonstrated significant potential for accurate contouring in MRgRT [4], [5], offering opportunities to improve treatment delivery and workflow efficiency. However, automatic delineation of the mesorectal CTV remains challenging, particularly at the craniocaudal boundaries, where anatomical variability and complexity often lead to inaccuracies [6], [7], [8]. The planning CTV, manually delineated in the patient simulation phase before treatment fractions, provides essential information about the target’s shape and craniocaudal extent. Integrating this information into deep learning models could enhance the accuracy of daily treatment contours. While both segmentation- and registration-based deep learning approaches can leverage the planning CTV, their relative effectiveness for mesorectal CTV contouring remains unclear.

This study compared deep learning-based segmentation and registration models for incorporating the planning contour to improve CTV contouring in online adaptive MRgRT. The methods’ accuracy, clinical usability, and robustness were evaluated to identify the optimal approach.

## 2 Materials and methods

### 2.1 Data and preprocessing

We collected a dataset of 107 intermediate-risk or locally advanced rectal cancer patients who underwent short-course radiotherapy (5 × 5.0 Gy) at the University Medical Center Utrecht, The Netherlands, between July 2020 and August 2024. The subjects gave informed consent as part of the prospective ethics review board-approved MOMENTUM study (NCT04075305) [9]. Each subject had a T2-weighted planning MRI on a 3T scanner (Ingenia, v5.4-7, Philips Medical Systems, The Netherlands) and was treated on a 1.5T MR-linac (Unity, Marlin v5.3-7, Elekta AB, Sweden) (Table 1) with an adapt-to-shape procedure [10]. The mesorectal CTV was manually delineated by a radiation oncologist on the planning MRI, which was then propagated to the fraction MRI using the Monaco treatment planning system (v5.40.01, Elekta AB, Sweden). The propagated contour was manually adjusted by a radiation oncologist or a radiotherapy technician if needed, obtaining the ground-truth mesorectal CTV contour.

**Table 1:** Dataset details and image acquisition parameters.

		Planning MRI	Daily fraction MRI
Subjects (train/val/test)		104 (68/14/22)	

Images (train/val/test)		68 (68/0/0)	543 (354/70/110)

Sequence		3D T2-weighted turbo spin-echo	3D T2-weighted turbo spin-echo

Magnetic field strength (T)		3	1.5
Echo time (ms)		270	120–124
Echo train length (N)		130	91–100
Repetition time (ms)		1577–5890	1300–1635
Bandwidth (Hz/px)		644	542–820

Acquisition matrix	Lower	308 x 308 x 150	268 x 268 x 83
	Upper	333 x 333 x 150	348 x 348 x 125

Slice thickness (mm)		2.0	2.0–3.0
In-plane voxel spacing (mm)		0.56–0.78	0.58–0.78
Image dimensions	Lower	672 x 672 x 150	528 x 528 x 83
	Upper	704 x 704 x 150	640 x 640 x 125

Data was split at the patient level into training (68 subjects), validation (15), and testing (24). The training set included the planning MRI and five daily fraction MRIs with their corresponding contours, while the validation and test sets consisted of the five daily fraction MRIs and the planning and fraction contours. Additionally, the training set contained 18 additional fraction MRIs with contours, available because motion between initial and position verification MRIs required recontouring, though four were missing due to data collection errors. MRIs from one validation subject and two test subjects were retrospectively excluded due to discrepancies between their planning and fraction CTV delineations, as confirmed by a radiation oncologist. In total, 422, 70, and 110 images/image pairs were used for training, validation, and testing.

#### Rigid registration.

The planning MRIs and their contours were registered to each daily MRI using iterative optimization in Elastix [11] (Section 1, Supplementary Material).

#### Image preprocessing.

For the segmentation model, images were resampled to a voxel spacing of 0.757×0.757×2.0mm using linear interpolation and intensity normalized using Z-score. During training, random patches of size 48 × 224 × 192 voxels were selected, with the patch size automatically configured (Section 2.2). The registration model used full field-of-view images, which were resampled to a voxel spacing of 2×2×2mm using linear interpolation, Z-score intensity normalized, and center-cropped, resulting in images of size 128 × 128 × 128. This image size improved registration over the use of patches with a higher in-plane resolution of 0.757 mm (Section 5.1, Supplementary Material).

### 2.2 Segmentation

**Fig. 1:**
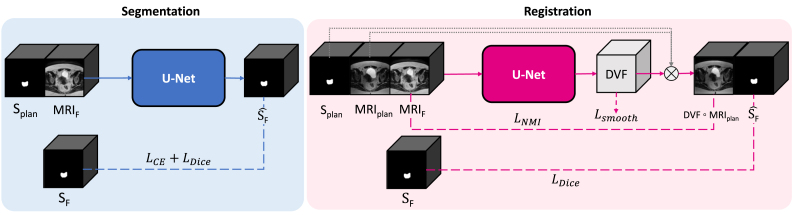
Overview of methods. The segmentation model receives S_plan_ and MRI_F_, and predicts Ŝ. The registration model receives S_plan_, MRI_plan_, and MRI_F_, and predicts the DVF, which propagates S_plan_ using image interpolation (⨂) to obtain Ŝ. The dashed lines indicate inputs that are used only during training and are not required during deployment. *MRI_plan_= planning MRI, MRI_F_= fraction MRI, S_plan_= planning segmentation, S=ground truth fraction segmentation, Ŝ= predicted segmentation*.

We used nnU-Net, a state-of-the-art deep learning segmentation framework, which automatically configures the network and training procedure based on the data and system specifications [12]. The model used two inputs: (1) the binary segmentation of the planning CTV (S_plan_) and (2) the fraction MRI (MRI_F_). It predicted a binary segmentation of the CTV (Ŝ_F_) (Fig. 1). During training, the ground truth fraction CTV (S_F_) was used in the objective function.

We used the 3D full-resolution U-Net with six encoder and decoder stages, each comprising two convolutional layers with [32, 64, 128, 256, 320, 320] filters, followed by LeakyReLU. It operated on a patch size of 48 × 224 × 192 voxels and contained 30.7 million trainable parameters.

The loss function combined cross-entropy loss ($L_{CE}$) and Soft-Dice loss ($L_{Dice}$) with a relative weight of 1. Deep supervision was applied at all decoder resolution levels [12]. Training was conducted on a PC with a 46 GB GPU (L40, Nvidia, USA), using a batch size of 2 and stochastic gradient descent (learning rate=0.01, weight decay=3e-5, momentum=0.99) for 1000 epochs (250 mini-batches each). Data augmentation included geometric (rotations, scaling, mirroring), intensity transformations (noise, blur, brightness, contrast) and low-resolution simulations, which are detailed in [12], [13]. The model predicted a binary segmentation map using a sliding window approach, followed by patch merging and keeping the largest connected component [12].

### 2.3 Registration

We used a 3D U-Net [14] with three inputs: (1) the binary segmentation of the planning CTV (S_plan_), (2) the planning MRI (MRI_plan_: moving), and (3) the fraction MRI (MRI_F_: fixed) (Fig. 1). The model predicted a displacement vector field (DVF) describing the spatial relationship between the planning and fraction MRIs. The DVF deformed the planning MRI and contour, aligning them with the fraction MRI and obtaining the fraction segmentation (S_F_ ^). During training, the ground truth fraction CTV (S_F_) was used in the objective function.

The U-Net had six encoder and decoder stages, each comprising two convolutional layers with [32, 64, 128, 256, 320, 320] filters and LeakyReLU, consistent with the segmentation model. We also explored a cascaded model with two U-Nets operating at half and full resolution, but found no significant improvement over a single U-Net (Section 5.2, Supplementary Material)

The loss function ($L_{reg}$; Eq. (1)) combined three weighted terms: (1) image similarity ($L_{NMI}$) based on normalized mutual information (NMI) between the deformed planning MRI and the fraction MRI; (2) the soft-Dice loss ($L_{Dice}$), to promote overlap between the deformed planning segmentation and fraction segmentation; and (3) regularization ($L_{smooth}$), the L2-norm of the DVF gradients, promoting smooth deformations. The first two terms were scaled by the factor λ, which was set to 0.4 (Section 5.3, Supplementary Material), and the regularization term was scaled by the regularization weight (RW), which was set to 5.

(1) $$L_{reg}=\left(1-\lambda \right)*L_{NMI}+\lambda *L_{Dice}+\text{RW}*L_{smooth}$$

The model was trained on a PC with a 46 GB GPU (L40, Nvidia, USA), using a batch size of 1 and the Adam optimizer (learning rate=0.0001). Convergence occurred after 120 epochs, each with 354 iterations (Section 3, Supplementary Material). The same data augmentations used for segmentation were applied to the registration model. At deployment, the predicted DVF with voxel spacings of 2×2×2mm was resampled to the original voxel spacing before propagating the planning contour to the fraction MRI.

### 2.4 Evaluation

The methods’ accuracy, clinical usability, and robustness against domain shifts were evaluated. Our code and models are publicly available.^1^ Supplementary Material (Section 2) details the software used in this study.

Contour accuracy was evaluated using the Dice similarity coefficient (Dice) and the maximum Hausdorff distance (HD) of the predicted contour and the ground truth of the mesorectal CTV. Additionally, the HD of the 30% most caudal, 40% middle and 30% most cranial slices evaluated the accuracy in different mesorectal zones.

The clinical usability of the contours was evaluated in ten single fractions of randomly selected test subjects (Section 6, Supplementary Material). The contours were provided to a single observer, a radiation oncologist with 13 years of experience, who was blinded to their origin. The observer scored using a five-point Likert scale [15]: 1. unusable, 2. major edits (user prefers to start from scratch), 3. minor edits necessary (user prefers not to start from scratch), 4. minor edits are not necessary (edits are stylistic and clinically unimportant), 5. use as-is. We considered scores 4 and 5 clinically acceptable.

Model robustness was evaluated as the accuracy under four simulated perturbations: (1) Rician noise in the fraction MRI, (2) Contrast modifications (gamma-transformations) in the fraction MRI, (3) Residual cranial translations (z) between fraction and planning, and (4) Rectal volume differences between fraction and planning. Each perturbation was applied to the test images at varying magnitude levels, generating 110 modified test images per perturbation level (see Section 4, Supplementary Material, for more details).

The developed segmentation (**Seg**) and registration (**Reg**) models were compared with baseline models (**SegBase** and **RegBase**), which did not utilize planning CTV information, relying solely on the fraction MRI for segmentation or the MRI pair for registration. We also included **clinical software** as benchmark (MIM Maestro, version 7.2.8, MIM Software Inc., USA), which propagated planning contours to the fraction through iterative registration (using free-form deformations and a multi-modality, gradient-based feature descriptor and regularization) [16]. The robustness of the clinical software was evaluated under a subset of perturbation levels to provide a reference baseline, with translational perturbations excluded due to the built-in rigid registration in the software.

Finally, the models were compared with a recent method that incorporates planning-based contours into segmentation models, which has shown improvements in mesorectal CTV contouring [17], [18], [19], [20]. The model, **SegBase-FT**, started as SegBase and was fine-tuned for each test subject using that subject’s planning MRI and contour (50 epochs; learning rate 0.0001). This approach simulated a personalized clinical workflow, using the planning MRI acquired in advance to personalize the model for subsequent treatment sessions. SegBase-FT is the set of 22 subject-specific models, one for each test subject.

Statistical significance was tested using two-way repeated measures ANOVA with two within-subject variables (method and fraction number) on rank-transformed data. We performed pairwise comparisons with Bonferroni correction using the Wilcoxon signed-rank test.

## 3 Results

**Fig. 2:**
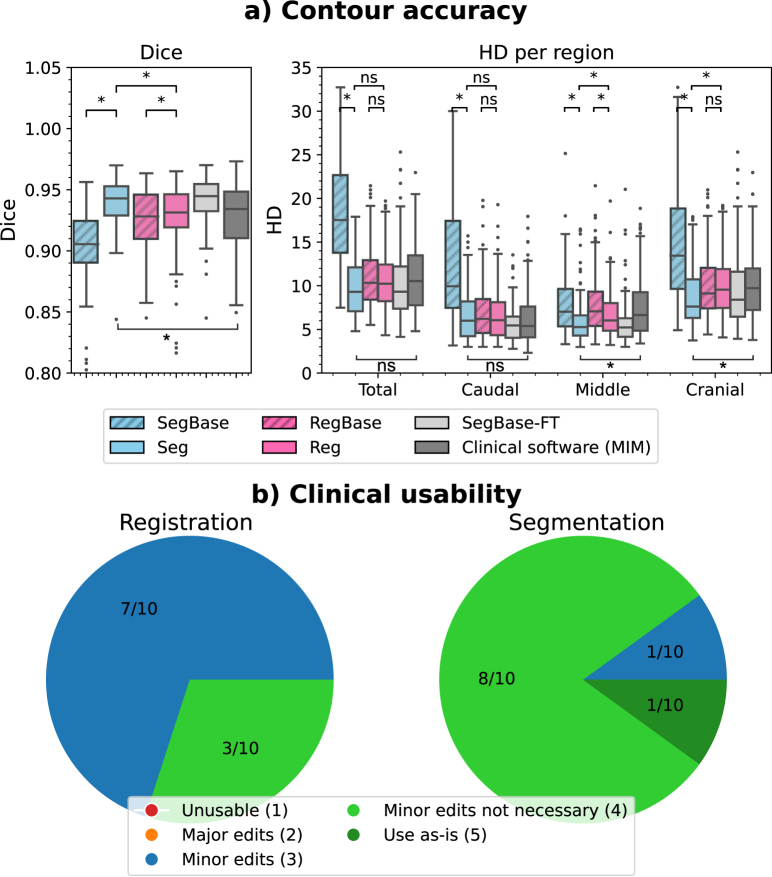
The contour accuracy of segmentation and registration models: **(a)** The median (IQR) Dice and HD (mm) per region in the test set. **(b)** The clinical usability score (five-point Likert scale) in ten single fractions in the test set.

**Fig. 3:**
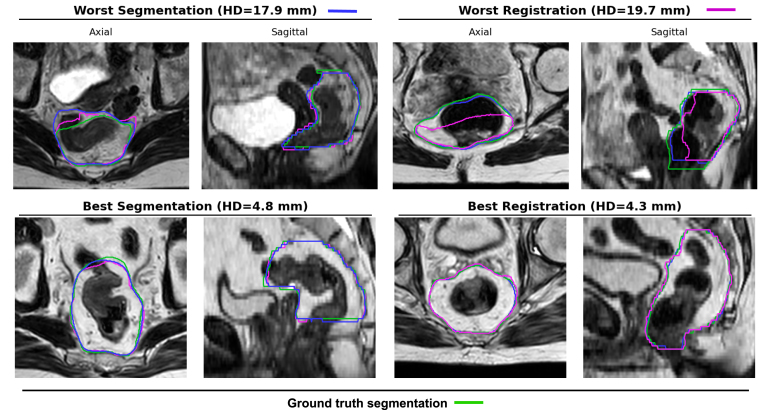
The ground truth (green) and predicted contours of the segmentation (blue) and registration (pink) methods.

The segmentation and registration models achieved median Dice scores of 0.94 (interquartile range, IQR: 0.93–0.95) and 0.93 (0.92–0.95), respectively (p<0.05), and HD values of 9.3 mm (7.1–12.1) and 10.2 mm (8.2–12.4) (p=0.18) (Fig. 2(a)). In the middle and cranial regions of the CTV, segmentation achieved significantly lower HD values than registration (HD_middle_: 5.3 mm (4.3–6.6) vs. 6.0 mm (4.8–8.0), p<0.05; HD_cranial_: 7.6 mm (6.3–10.7) vs. 9.6 mm (7.5–11.9), p<0.05) (Fig. 2(a); Fig. 3). In addition, segmentation scored higher in clinical usability, with 9/10 contours rated as clinically acceptable (score>=4), compared to 3/10 for registration (Fig. 2(b)).

Incorporating the planning CTV improved the performance of both models. For segmentation, the HD reduced from 17.5 mm (13.7–22.6) in SegBase to 9.3 mm (7.1–12.1) in Seg (p<0.05). For registration, the improvements were limited to the middle region, reducing HD_middle_ from 7.1 mm (5.3–9.3) in RegBase to 6.0 mm (4.8–8.0) in Reg (p<0.05).

The segmentation model outperformed clinical software, with a higher Dice score (0.94 [IQR: 0.93–0.95] versus 0.93 [0.91–0.95], p<0.05) and lower HD_middle_ (5.3 mm ([4.3–6.6] vs. 6.6 mm (4.8–9.3), p<0.05) and lower HD_cranial_ values (7.6 mm [6.3–10.7] vs. 9.7 mm [7.2–12.0], p<0.05). Registration achieved accuracies similar to that of clinical software (p>0.2, all metrics). In addition, segmentation performed comparably to SegBase-FT (p>0.8, all metrics), whereas registration slightly underperformed SegBase-FT (p<0.05, all metrics except HD_total_ and HD_cranial_).

**Fig. 4:**
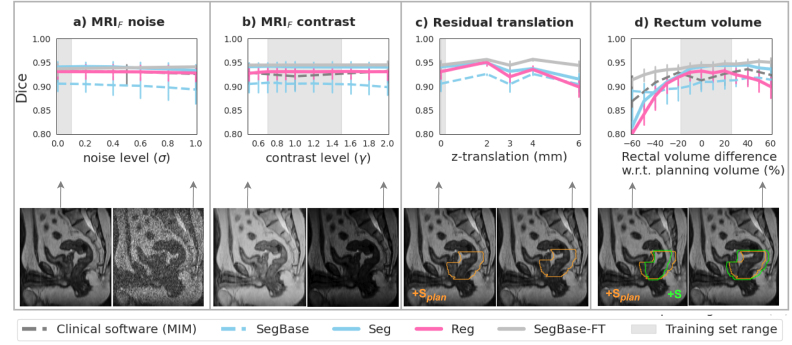
The effect of simulated variations in: **(a)** MRI noise, **(b)** MRI contrast, **(c)** Residual translations between MRI_plan_/S_plan_ and MRI_F_, and **(d)** Rectum volume. Lines and error bars show the median and IQR Dice in the test set. The semitransparent gray boxes indicate the training set range of noise and contrast variations (from data augmentation) and rectum volume variations (naturally occurring). *MRI_plan_=planning MRI; S_plan_=planning segmentation; MRI_F_=fraction MRI; S_F_=ground truth segmentation*.

Both models showed similar robustness to noise and contrast variations and residual translations (Fig. 4(a–c)), though segmentation was slightly more robust than registration against rectal volume differences between fraction and planning (Fig. 4(d)). However, the segmentation model’s robustness was not flawless, e.g., the Dice score dropped from 0.94 (0.93–0.95) to 0.91 (0.89–0.92) with 30% rectal emptying, slightly lower than that of clinical software. Similarly, the Dice score dropped for 6 mm (2 slices) translations. Visual examples of these failure cases are shown in Figure S3 (Section 8, Supplementary Material). Notably, SegBase-FT outperformed all other models in terms of robustness across all tested perturbations. In addition, SegBase outperforms Seg in extreme rectal emptying and filling.

Model inference was 27.3±7.8 s for segmentation and 0.4 ±0.3 s for registration (per image on the GPU).

## 4 Discussion

This study compared deep learning-based segmentation and registration models to identify the most effective method for incorporating planning contours into online mesorectal CTV contouring in MRgRT for rectal cancer. Incorporating planning contours as prior information improved performance for both models, with segmentation showing the largest improvements compared to its baseline. Segmentation slightly outperformed registration in the mid and cranial regions and produced more clinically acceptable contours, making it the preferred approach.

The improvements from using planning contours were substantial (Segmentation HD: 17.5→9.3mm; Registration HD_middle_: 7.1→6.0mm), and comparable to other strategies that leverage prior knowledge, such as through fine-tuning on individual patient data [17], [18], [19], [20] (as reflected by our comparison to SegBase-FT), patient-specific weight maps based on population statistics (mean surface distance, MSD: 4→2.5mm) [21] or multi-task learning (MSD: 3.4→1.8mm) [22]. Similar benefits have been observed in other MRgRT domains, such as abdominal cancers [23].

From a clinical perspective, the proposed method offers several advantages over these alternatives. Our approach integrates prior knowledge directly, without retraining, statistical modeling, or architectural changes, making it simpler to implement. Planning contours are routinely available at treatment and can be rigidly registered to the daily MRI as part of standard MRgRT workflows. Importantly, the resulting segmentation model’s accuracy in in-distribution data (median Dice: 0.94, IQR: 0.93–0.95) was comparable to interobserver agreement (interobserver values of 0.91 [8] and 0.94 [24]).

However, important challenges remain. The cranial region was particularly difficult to delineate (HD_cranial_: 7.6 mm, IQR: 6.3–10.7), slightly exceeding interobserver variability (6 mm, IQR: 4–8) [8]. In addition, the approach was counterproductive in cases with extreme anatomical variation (rectal emptying), which is precisely when adaptive MRgRT is most critical. In these cases, incorporating planning contours reduced segmentation accuracy, likely due to bias introduced by the contours themselves. These extreme variations were not represented in the training data, suggesting that expanding the dataset to include such cases could improve model robustness. While patient-specific fine-tuning improved performance in these challenging conditions, it is not clinically feasible due to its resource-intensive nature. These findings indicate significant room for improvement and underscore the importance of evaluating performance under challenging anatomical scenarios.

The difference in clinical acceptability between the registration and segmentation tasks may be attributed to the editing effort required. While both typically involved minor adjustments, registration edits were more clinically significant and were estimated to require more time, particularly in ventral regions. In contrast, segmentation errors, especially at the cranial and caudal boundaries, were easier to correct, as they predominantly involved over-segmentation. For instance, 70% of segmentation predictions exceeded (over-segmented) the caudal boundary (compared to only 9% under-segmented), often requiring just a simple deletion. In comparison, the registration model over-segmented the caudal boundary in 51% and under-segmented it in 20% of cases.

This study has several limitations. First, the relatively small sample size may limit generalizability. However, it is consistent with the scale of most studies in this field [8], [20], [25], [26]. We therefore believe the findings are representative of the current clinical landscape of MRgRT. Second, the qualitative evaluation of clinical usability was based on only ten subjects and should be viewed as supportive rather than conclusive. Finally, the segmentation and registration models used different input sizes and in-plane resolutions (0.757 mm vs. 2 mm), which may limit direct comparison. Nonetheless, the lower resolution yielded better registration results (see Section 5.1, Supplementary Material), and both methods were independently optimized, allowing a fair comparison of the best-performing versions of both methods.

We emphasize the importance of evaluating the generalizability of deep learning models across diverse clinical settings. Our robustness experiments demonstrated that the segmentation model incorporating prior information achieved performance comparable to inter-observer agreement. However, when tested on data with slightly different characteristics, such as increased anatomical variation between planning and fraction scans, the model without prior information demonstrated greater robustness. To support the development of safe and reliable deep learning models, future research should place greater emphasis on evaluating robustness under varying conditions. These include differences in scanners, imaging protocols, subject demographics, and treatment protocols that influence anatomical variability.

In conclusion, integrating the planning contour into deep learning-based segmentation and registration models improves online mesorectal CTV contouring in MRgRT for rectal cancer. Segmentation was identified as the best approach among segmentation and registration. However, substantial challenges remain, particularly in cases with extreme rectal filling differences, where model performance declines. This work advances the field of MRgRT by comparing different approaches for mesorectal CTV contouring, investigating their potential for clinical implementation.

## CRediT authorship contribution statement

**Iris D. Kolenbrander:** Writing – original draft, Conceptualization, Metholodogy, Software, Validation, Formal analysis, Investigation, Visualization. **Koen M. Kuijer:** Metholodogy, Software, Validation, Investigation, Data curation. **Mark H.F. Savenije:** Data curation. **Gert J. Meijer:** Conceptualization, Writing – review & editing. **Martijn P.W. Intven:** Data curation, Conceptualization. **Josien P.W. Pluim:** Supervision, Writing – review & editing, Conceptualization. **Matteo Maspero:** Writing – review & editing, Supervision, Methodology, Investigation, Conceptualization.

## Declaration of Generative AI and AI-assisted technologies in the writing process

During the preparation of this work the authors used Microsoft Copilot and DeepL Write to improve the readability and conciseness of the manuscript. After using these tools, the authors reviewed and edited the content as needed and take full responsibility for the content of the publication.

## Funding

The Irene Curie Fellowship program and the Eindhoven Artificial Intelligence Systems Institute supported this work.

## Declaration of competing interest

The authors declare the following financial interests/personal relationships which may be considered as potential competing interests: Martijn P. W. Intven reports financial support from Elekta AB, including speaking and lecture fees, and grants from the Dutch Cancer Society. He is a board member at the Dutch Society for Radiotherapy and Oncology.

## Appendix A Supplementary data

MMC S1 Additional findings.

## Footnotes

1 Code repository: https://github.com/iriskolenbrander/Conditional_SegReg.git.

## References

[1] Intven M.P.W., de Mol van Otterloo S.R., Mook S., Doornaert P.A.H., de Groot-van Breugel E.N., Sikkes G.G., Willemsen-Bosman M.E., van Zijp H.M., Tijssen R.H.N. (2021). Online adaptive MR-guided radiotherapy for rectal cancer; feasibility of the workflow on a 1.5T MR-linac: clinical implementation and initial experience. Radiother Oncol.

[2] Güngör G., Serbez İ., Temur B., Gür G., Kayalılar N., Mustafayev T., Korkmaz L., Aydın G., Yapıcı B., Atalar B., Özyar E. (2021). Time analysis of online adaptive magnetic resonance–guided radiation therapy workflow according to anatomical sites. Pr Radiat Oncol.

[3] Pierrard J., Heylen S., Vandermeulen A., Van Ooteghem G. (2024). Dealing with rectum motion during radiotherapy: How can we anticipate it?. Tech Innov Patient Support Radiat Oncol.

[4] Xia S., Li Q., Zhu H.T., Zhang X.Y., Shi Y.J., Yang D., Wu J., Guan Z., Lu Q., Li X.T. (2024). Fully semantic segmentation for rectal cancer based on post-nCRT MRI modality and deep learning framework. BMC Cancer.

[5] Kolenbrander I.D., Maspero M., Hendriksen A.A., Pollitt R., van der Voort van Zyp J.R.N. (2024). Deep-learning-based joint rigid and deformable contour propagation for magnetic resonance imaging-guided prostate radiotherapy. Med Phys.

[6] White I., Hunt A., Bird T., Settatree S., Soliman H., Mcquaid D., Dearnaley D., Lalondrelle S., Bhide S. (2021). Interobserver variability in target volume delineation for CT/MRI simulation and MRI-guided adaptive radiotherapy in rectal cancer. Brit J Radiol.

[7] Valentini V., Gambacorta M.A., Barbaro B., Chiloiro G., Coco C., Das P., Fanfani F., Joye I., Kachnic L., Maingon P., Marijnen C., Ngan S., Haustermans K. (2016). International consensus guidelines on clinical target volume delineation in rectal cancer. Radiother Oncol.

[8] Ferreira Silvério N., van den Wollenberg W., Betgen A., Wiersema L., Marijnen C., Peters F., van der Heide U.A., Simões R., Janssen T. (2024). Evaluation of deep learning clinical target volumes auto-contouring for magnetic resonance imaging-guided online adaptive treatment of rectal cancer. Adv Radiat Oncol.

[9] de Mol van Otterloo S., Christodouleas J., Blezer E., Akhiat H., Brown K., Choudhury A., Eggert D., Erickson B., Faivre-Finn C., Fuller C. (2020). The MOMENTUM study: an international registry for the evidence-based introduction of MR-guided adaptive therapy. Front Oncol.

[10] Winkel D., Bol G.H., Kroon P.S., van Asselen B., Hackett S.S., Werensteijn-Honingh A.M., Intven M.P., Eppinga W.S., Tijssen R.H., Kerkmeijer L.G. (2019). Adaptive radiotherapy: the elekta unity MR-linac concept. Clin Transl Radiat Oncol.

[11] Klein S., Staring M., Murphy K., Viergever M., Pluim J. (2009). Elastix: a toolbox for intensity-based medical image registration. IEEE Trans Med Imaging.

[12] Isensee F., Jaeger P.F., Kohl S.A., Petersen J., Maier-Hein K.H. (2021). nnU-Net: a self-configuring method for deep learning-based biomedical image segmentation. Nat Methods.

[13] Isensee F., Jäger P., Wasserthal J., Zimmerer D., Petersen J., Kohl S., Schock J., Klein A., Roß T., Wirkert S. (2020). Batchgenerators — a python framework for data augmentation. Zenodo.

[14] Ronneberger O., Fischer P., Brox T. (2015). Med image comput comput-assisted intervent.

[15] Kraus A.C., Iqbal Z., Cardan R.A., Popple R.A., Stanley D.N., Shen S., Pogue J.A., Wu X., Lee K., Marcrom S., Cardenas C.E. (2024). Prospective evaluation of automated contouring for CT-based brachytherapy for gynecologic malignancies. Adv Radiat Oncol.

[16] Piper J., Richmond J., Nelson A. (2018). https://go.mimsoftware.com/hubfs/VoxAlign_Deformation_Engine_White_Paper.pdf.

[17] Elmahdy M.S., Ahuja T., van der Heide U.A., Staring M. (2020). International symposium on biomedical imaging.

[18] Li Z., Zhang W., Li B., Zhu J., Peng Y., Li C., Zhu J., Zhou Q., Yin Y. (2022). Patient-specific daily updated deep learning auto-segmentation for MRI-guided adaptive radiotherapy. Radiother Oncol.

[19] Smolders A., Lomax A., Weber D.C., Albertini F. (2023). Patient-specific neural networks for contour propagation in online adaptive radiotherapy. Phys Med Biol.

[20] Kensen C.M., Simões R., Betgen A., Wiersema L., Lambregts D.M., Peters F.P., Marijnen C.A., van der Heide U.A., Janssen T.M. (2024). Incorporating patient-specific information for the development of rectal tumor auto-segmentation models for online adaptive magnetic resonance image-guided radiotherapy. Phys Imaging Radiat Oncol.

[21] Ferreira Silvério N., van den Wollenberg W., Betgen A., Wiersema L., Marijnen C.A., Peters F., van der Heide U.A., Simões R., Intven M.P., van der Bijl E., Janssen T. (2025). Incorporating patient-specific prior clinical knowledge to improve clinical target volume auto-segmentation generalisability for online adaptive radiotherapy of rectal cancer: A multicenter validation. Radiother Oncol.

[22] Elmahdy M.S., Beljaards L., Yousefi S., Sokooti H., Verbeek F., Van Der Heide U.A., Staring M. (2021). Joint registration and segmentation via multi-task learning for adaptive radiotherapy of prostate cancer. IEEE Access.

[23] De Benetti F., Delopoulos N., Belka C., Corradini S., Navab N., Wendler T., Albarqouni S., Landry G., Kurz C. (2025). Enhancing patient-specific deep learning based segmentation for abdominal magnetic resonance imaging-guided radiation therapy: A framework conditioned on prior segmentation. Phys Imaging Radiat Oncol.

[24] White I., Hunt A., Bird T., Settatree S., Soliman H., Mcquaid D., Dearnaley D., Lalondrelle S., Bhide S. (2021). Interobserver variability in target volume delineation for CT/MRI simulation and MRI-guided adaptive radiotherapy in rectal cancer. Br J Radiol.

[25] Li J., Song Y., Wu Y., Liang L., Li G., Bai S. (2023). Clinical evaluation on automatic segmentation results of convolutional neural networks in rectal cancer radiotherapy. Front Oncol.

[26] Geng J., Zhang S., Wang R., Bai L., Chen Q., Wang S., Zhu X., Liu Z., Yue H., Wu H., Li Y., Du Y. (2024). Deep-learning based triple-stage framework for MRI-CT cross-modality gross tumor volume (GTV) segmentation for rectal cancer neoadjuvant radiotherapy. Biomed Signal Process Control.

