# Supplementary material for: Comparison of deep learning-based segmentation and registration using pre-treatment contours for online rectal delineation in magnetic resonance-guided radiotherapy
Source: Phys Imaging Radiat Oncol. 2025 Oct 21;36:100854. doi: 10.1016/j.phro.2025.100854 (PMC12590434; doi:10.1016/j.phro.2025.100854)
Supplement: MMC S1 — Additional findings. [file mmc1.pdf]

Supplementary materials

Of *"Comparison of deep learning-based segmentation and registration using pre-treatment contours for online rectal delineation in magnetic resonance-guided radiotherapy." Iris D. Kolenbrander, Koen Kuijer, Mark Savenije, Gert Meijer, Martijn Intven, Josien P.W. Pluim, and Matteo Maspero*

Contents

1 Iterative rigid registration **2**

2 List of software **2**

3 Model convergence **3**

4 Simulated perturbations **3**

5 Registration: Method development **4**

5.1 Input resolution 4

5.2 Architecture 5

5.3 Learning strategy 5

6 Subset for clinical usability assessment **6**

7 Added path length (APL) **6**

8 Failure examples under simulated perturbations **7**

1 Iterative rigid registration

The planning MRI was registered to each daily MRI in Elastix using normalized mutual information (NMI) as the objective function and stochastic gradient descent in a multiresolution registration scheme[1]. The registration was performed over four resolution levels, with isotropic downsampling factors of 8, 4, 2, and 1, and the optimization was run for 2000 iterations per level.

The hyperparameter a, related to the optimizer's step size in stochastic gradient descent, was set to 2000 in the original parameter file[1]. We experimented with values of 1000 and 5000, which achieved Dice values of 0.90 (0.88-0.93) and 0.86 (0.83-0.90), compared to 0.91 (0.88-0.93) for 2000, and proceeded with a value of 2000. In addition, we experimented with adaptive stochastic gradient descent, which estimates an appropriate value for each image pair. However, this resulted in a lower median Dice in the validation set of 0.89 (interquartile range, IQR: 0.85-0.92) compared to 0.91 (0.88-0.93) for a fixed a value of 2000.

2 List of software

Table S1: Main software and packages used in this study

**Software/Package/Library**	**Version**	**Purpose**
Elastix	5.0.1	Rigid image registration
Anaconda	25.1.0	Python environment and package management
Python	3.10.15	Image preprocessing, model development, and evaluation
Torch	2.5.1	Model training and inference
Pytorch Lightning	2.5.0	Model training and inference
Torchvision	0.20.1	Data loading and augmentation for model training
nnU-Net	v2	Segmentation model training
SimpleITK	2.4.1	Image preprocessing
Monai	1.3.2	Evaluation
Pandas	2.2.3	Evaluation
Seaborn	0.13.2	Visualization
Scipy	1.15.1	Statistical analysis

[1]Parameter file: https://github.com/SuperElastix/ElastixModelZoo/blob/master/models/Par0001/Par0001rigid.txt

3 Model convergence

A notable difference between the deep learning-based segmentation and registration models is their training duration: the segmentation model converged in 970 epochs (970*250=242,500 iterations; batch size = 2 image patches), whereas the registration model required only 115 training epochs (115*354=40,710 iterations; batch size = 1 image pair). The segmentation model's weights were saved solely at the final epoch (as per the default in nnU-Net), while the registration model's weights were saved every 10 epochs. Consequently, we selected the 1000th epoch for segmentation and the 120th epoch for registration. The discrepancy in convergence speed may be attributed to task differences and the input FOV.

Convergence was verified using validation Dice curves, with a stopping criterion based on the running average and standard deviation of the Dice over a 15-epoch window (spanning three monitored epochs, as the Dice was monitored every 5 epochs). The stopping epoch was defined as the point when both the running average's change and the standard deviation dropped below their respective thresholds (0.0001 and 0.002) for 20 consecutive epochs, indicating stable model performance. For transparency, the validation curves during training are shown in Figure S1.

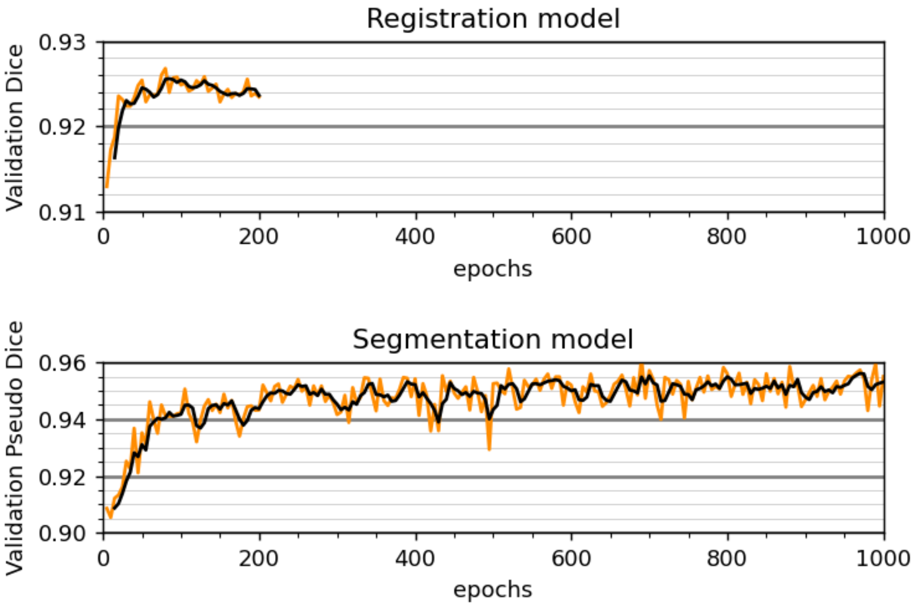

Figure S1: Validation (pseudo) Dice curves (orange) during training of the segmentation and registration model and their running average over 15 epochs (black).

4 Simulated perturbations

Noise in $\mathbf{MRI_F}$: Rician noise was added to each fraction MRI (MRI_F) using the implementation of MONAI [1]. Specifically, two independent Gaussian noise components, N_1 and N_2, were added. The distributions were sampled independently from a Gaussian distribution $\mathcal{N}(0, \sigma^2_{\text{noise}})$, where σ_{noise} represents the noise's standard deviation and determines the perturbation's magnitude. The value of σ_{noise} ranged from 0 to 1.0 (step=0.1) and was scaled relative to the standard deviation of the image intensities. The perturbed image MRI'_F at each pixel x is computed as:

$$MRI'_F(x) = |MRI_F(x) + N_1(x) + iN_2(x)| \tag{1}$$

Note that Gaussian noise was added to the training images as part of data augmentation (σ_{noise}=0.1).

Contrast in $\mathbf{MRI_F}$: Gamma transformations were applied to modify fraction MRI intensities using the implementation of MONAI [1]. To lighten the image, γ ranged from 0.5 to 1.0 (step=0.1), and to

darken it, from 1.0 to 2.0 (step=0.2), affecting the image contrast (relative difference between structures). The perturbed image MRI'_F at each pixel x is computed as:

$$MRI'_F(x) = MRI_F(x)^{\gamma} \tag{2}$$

Note that gamma transformations were also applied to the training images as part of data augmentation (γ ranged between 0.7 and 1.5).

Residual translation: Cranial (z) translations were applied to the planning MRI and contour to simulate suboptimal rigid registration. We focused solely on cranial translations, rather than including rotations or translations in other directions, because these were the largest misalignments observed between the planning and online fraction MRIs in the training set (Table S2). Translations ranged from 0 to 6 mm in steps of 2 or 3 mm (i.e., 1 slice per step).

Table S2: Rigid parameters in the training set.

**Statistic**	**Translation (mm)**			**Rotation (degrees)**		
	**x**	**y**	**z**	**x**	**y**	**z**
**min**	-39	-31	-67	-3.8	-4.2	-3.3
**max**	31	21	82	5.0	4.2	3.6
**median**	-1	-15	0	0	0	0.1
**IQR**	[-6; 5]	[-16; -14]	[-16; 24]	[-0.6; 0.7]	[-0.6; 1.1]	[-0.5; 0.6]

Rectal volume: Rectal volume variations were simulated in the fraction MRIs and their corresponding contours by applying nonlinear, anatomy-informed spatial transformations as described in [2]. For each fraction MRI and its associated segmentation map $S(x, y, z)$, a displacement vector field (DVF) was generated by computing the gradient of the map after Gaussian smoothing:

$$DVF = \Delta(G * S(x, y, z)) \cdot C \tag{3}$$

Here, G denotes a Gaussian kernel used to smooth the segmentation map, and the scalar C controls the deformation amplitude, ranging from -2000 (rectal emptying) to +2000 (filling) in steps of 500.

The planning MRI and its contour remained undeformed, thereby introducing a rectal volume mismatch between the planning and fraction MRIs. The resulting differences in rectal volume ranged from -60% (emptying) to +60% (filling), relative to the planning rectal volume. Daily fraction MRIs with rectal volumes outside the 100-450 mL range were excluded from analysis.

5 Registration: Method development

Two additional metrics alongside Dice and HD were used to assess registration accuracy during method development (reported exclusively in the Supplementary Material). These included the structural similarity index measure (SSIM) of the planning MRI and fraction MRI and the percentage of negative Jacobian determinant values of the DVF. The latter indicates tissue folding. The registration model's input resolution, architecture, and learning strategy were optimized.

5.1 Input resolution

The original in-plane resolution of 0.757 mm, consistent with that of the segmentation model, was compared to an in-plane resolution of 2 mm, which matched the slice thickness and made the voxels isotropic. The original resolution images were center-cropped to 96 × 224 × 192, double the size of the segmentation model's patches (48 × 224 × 192), ensuring that the mesorectal CTV was fully visible in the input images. The images with lower in-plane resolution (2 mm) were resampled using linear interpolation and center-cropped to the size of 128 × 128 × 128.

The original resolution (0.757 mm) resulted in significantly lower contour accuracies compared to the lower resolution (2 mm), with median validation HD values of 11.6 mm (IQR: 9.0-14.8) and 11.0 mm (8.6-14.5), respectively ($p<0.05$, Figure S2a). This finding may be explained by the anisotropic voxel spacings off the original input. For each model, we optimized the learning rate and regularization weight

within the ranges [0.00005, 0.0001, 0.0005] and [1, 2, 5, 10], respectively, finding the optimal values of 0.0001 for the learning rate and 5 for the regularization weight.

5.2 Architecture

Since recent work has demonstrated that multi-resolution, cascaded models often perform better than their single-model counterparts [3–5], we compared the single U-Net to a cascaded model with two U-Nets operating at half and full resolution. In addition, we compare against a lightweight U-Net, commonly referred to as VoxelMorph [6]. We found no significant improvement of these models over the single U-Net, with a median HD of 10.1 mm (IQR: 8.4-13.9) and achieved by the two-level U-Net, 11.2 (IQR: 8.6-14.4) by VoxelMorph, and 11.0 mm (IQR: 8.6-14.5) by the single U-Net (p=0.1) (Figure S2b).

5.3 Learning strategy

Continuing with the in-plane resolution of 2 mm, we optimized the segmentation-loss weight among values of 0 (unsupervised), 0.2, 0.4, 0.6 (hybrid supervision), and 1.0 (weak supervision). Hybrid supervision (λ of 0.4) resulted in higher contour accuracies than unsupervised learning (HD: 9.6 mm [IQR: 7.6-12.7] vs. 11.0 mm [8.6-14.5]) and weak supervision (0.93 [0.91-0.94] vs. 0.92 [0.90-0.94]) ($p<0.05$, Figure S2c).

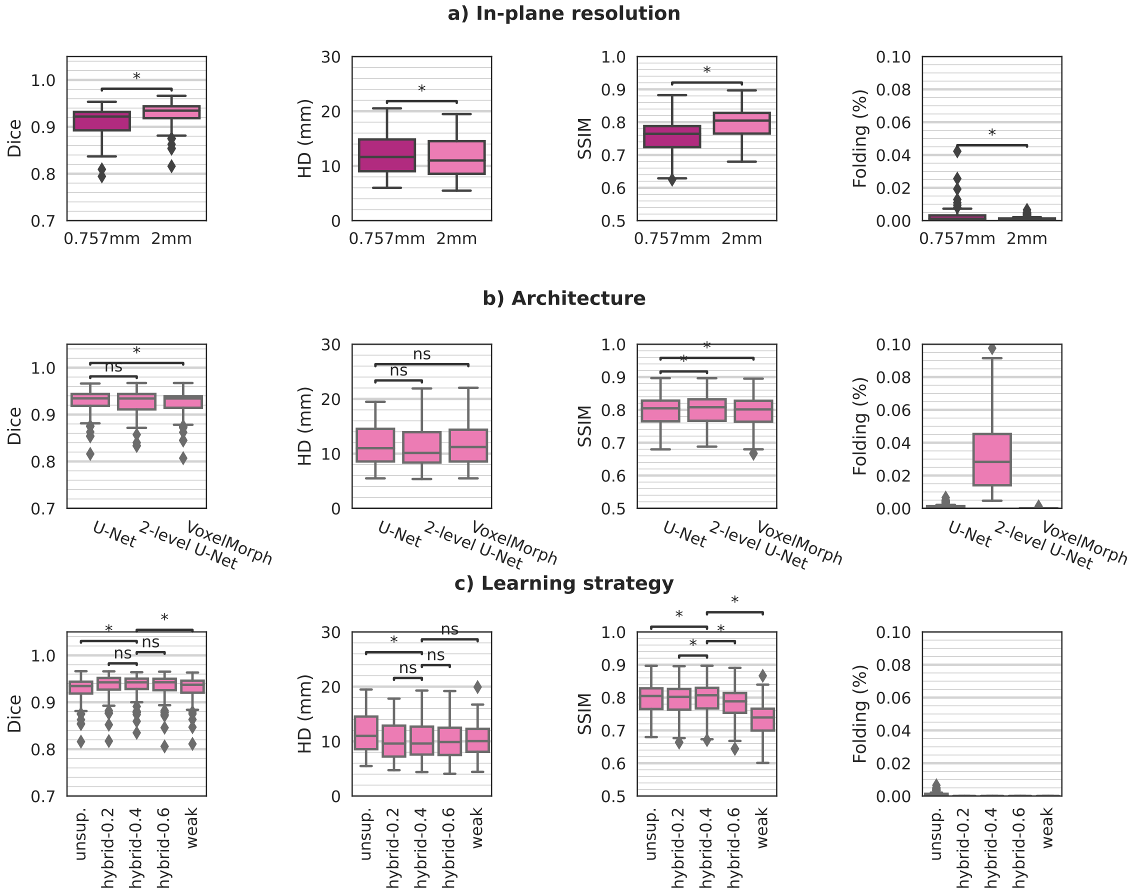

Figure S2: Comparison of different in-plane resolutions, architecture configurations, and learning strategies on the registration accuracy (Dice, HD, SSIM, and Folding) in the validation set. The different learning strategies contain different segmentation-loss weights with values of 0 (unsupervised), 0.2, 0.4, 0.6 (hybrid supervision), and 1.0 (weak supervision).

6 Subset for clinical usability assessment

We assessed model performances on the subset used for clinical usability assessments, containing single fractions of 10 randomly selected subjects, and on the complete test set. The registration model showed larger HD values in both datasets (Table S3), with median paired differences (Reg-Seg) of +1.2 mm (min-max range: -5.6 to 5.6 mm) in the subset and +0.9 mm (-11.7 to 11.3 mm) in the complete test set. These results confirm that the subset selected for qualitative evaluation represent the overall test set.

Table S3: Performance of models on all test subjects (N=22) and the subset (N=10) used for qualitative evaluation. Values are the median (min-max range) HD in mm.

**HD (mm)**	**Complete test set**	**Subset**
**Segmentation**	9.5 (4.8; 28.5)	10.4 (6.0; 16.7)
**Registration**	10.3 (4.3; 21.7)	11.6 (6.9; 16.2)
**Paired difference (Reg - Seg)**	+0.9 (-11.7; 11.3)	+1.2 (-5.6; 5.6)

7 Added path length (APL)

In addition to the primary metrics for evaluating contour accuracy, we also computed the added path length (APL) using the PlatiPy library [7]. APL quantifies the additional contour length that must be added to the predicted segmentation to match the reference (ground truth) contour. It is calculated slice-by-slice and then summed over all slices. This metric has been shown to correlate with manual editing time [8] and might be a valuable indicator of clinical usability. As shown in Figure S3, the segmentation method yielded slightly lower APL values than the registration method (median APL: 339 mm [IQR: 208-481] vs. 460 mm [IQR: 293-678]; p<0.05), consistent with the other findings in the main manuscript regarding contour accuracy.

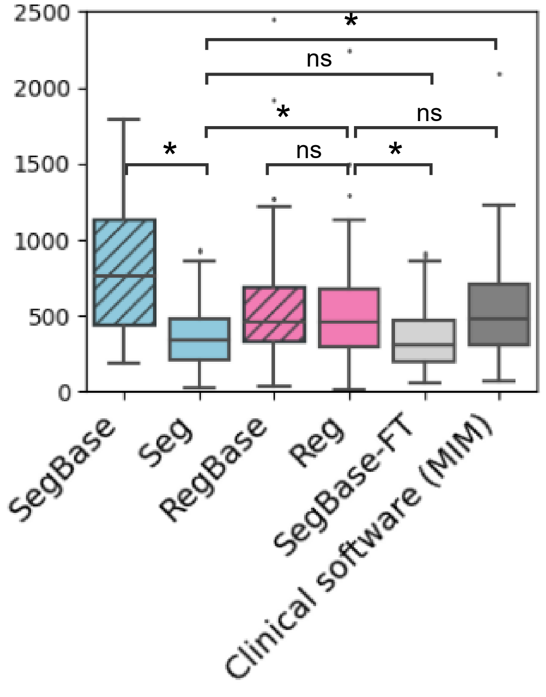

Figure S3: Added Path Length (APL) comparison between the segmentation and registration methods in the test set. Values are the median (IQR) of the slice-wise APL (mm) (summed over all slices).

8 Failure examples under simulated perturbations

Figure S4 presents representative examples of segmentation behavior under simulated perturbations. In Figure S4a, residual translations caused by inaccurate rigid registration lead to misalignments that shift the predicted CTV. As a result, the segmentation model over-segments the CTV in the caudal region and under-segments it cranially, with both the segmentation and registration models contributing to the cranial under-segmentation. Figures S4b–c illustrate cases with rectal volume differences compared to the planning scan. When the rectum is emptied, both models tend to over-segment the CTV, which aligns with expectations given the larger rectal volume used as prior information. Conversely, in cases of rectal filling, the registration model slightly under-segments the CTV in some areas, while the segmentation model often still captures the full structure. This suggests that the segmentation model is capable of generalizing beyond the provided prior label, even when the anatomy deviates from the planning conditions.

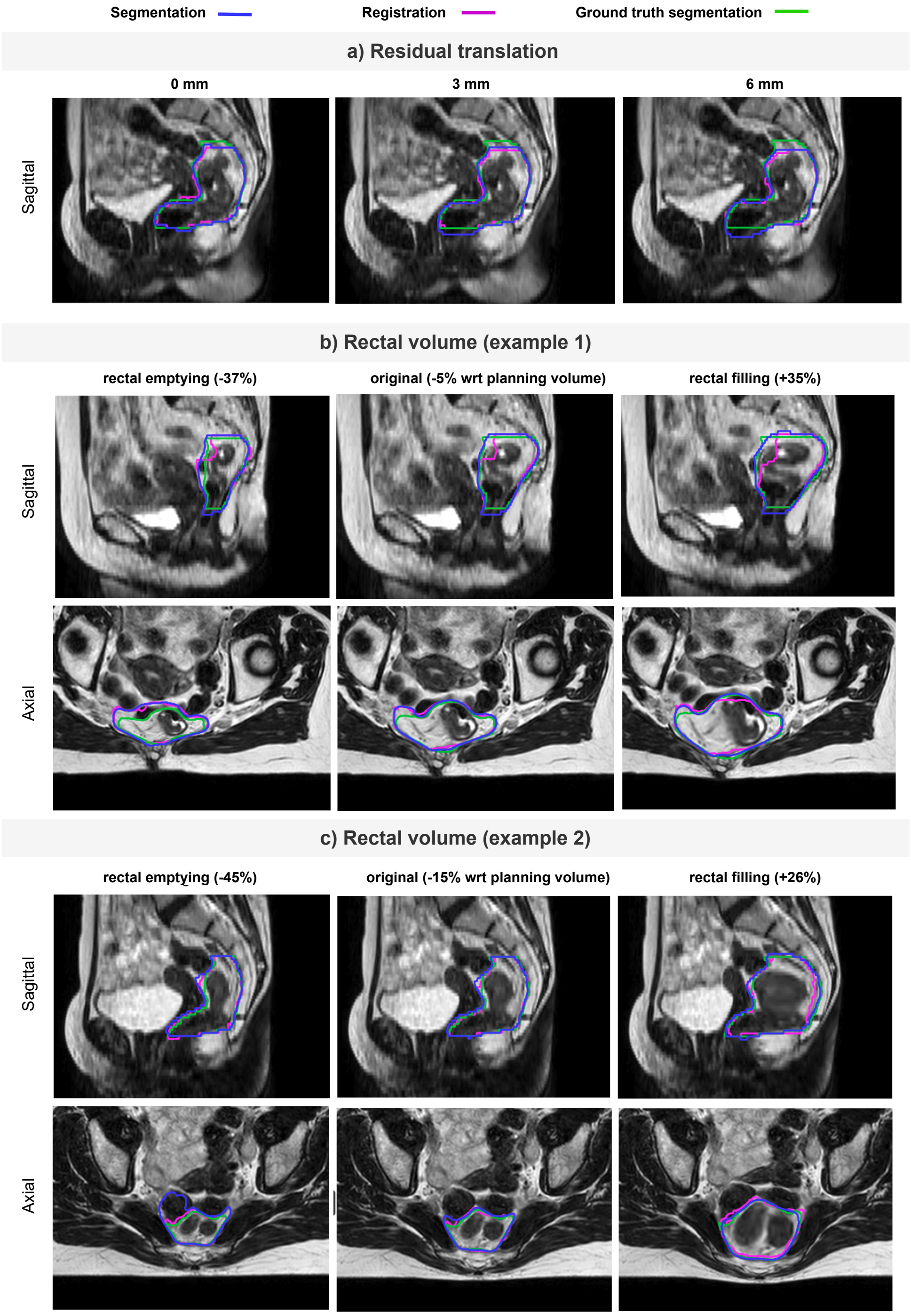

Figure S4: Representative examples of failure modes under simulated perturbations. **(a)** Case showing residual translational misalignment between MRI_{plan}/S_{plan} and MRI_F. **(b, c)** Cases illustrating rectal volume differences (between S_F and S_{plan}). *MRI_{plan}=planning MRI; S_{plan}=planning segmentation; MRI_F=fraction MRI; S_F=ground truth segmentation*

References

[1] Cardoso, M. J., Li, W., Brown, R., Ma, N., Kerfoot, E., Wang, Y., Murrey, B., Myronenko, A., Zhao, C., Yang, D., et al., MONAI: An open-source framework for deep learning in healthcare, arXiv preprint arXiv:2211.02701 (2022).

[2] Kovacs, B., Netzer, N., Baumgartner, M., Eith, C., Bounias, D., Meinzer, C., Jäger, P. F., Zhang, K. S., Floca, R., Schrader, A., et al., Anatomy-informed data augmentation for enhanced prostate cancer detection, in: Med Image Comput Comput-Assisted Intervent (MICCAI), Springer, 2023, pp. 531–540. https://doi.org/10.1007/978-3-031-43990-2_50.

[3] Mok, T., Chung, A., Large deformation diffeomorphic image registration with laplacian pyramid networks., in: Med Image Comput Comput-Assisted Intervent (MICCAI), Springer, 2020, pp. 211–221. https://doi.org/10.1007/978-3-030-59716-0_21.

[4] Kolenbrander, I. D., Maspero, M., Hendriksen, A. A., Pollitt, R., Zyp, J. R. N.van der Voort van, et al., Deep-learning-based joint rigid and deformable contour propagation for magnetic resonance imaging-guided prostate radiotherapy, Med Phys 51 (2024) 2367–2377. https://doi.org/10.1002/mp.17000.

[5] Tian, L., Greer, H., Kwitt, R., Vialard, F.-X., San José Estépar, R., et al., UniGradICON: a foundation model for medical image registration, in: Linguraru, M. G., Dou, Q., Feragen, A., Giannarou, S., Glocker, B., et al. (Eds.), Med Image Comput Comput-Assisted Intervent (MICCAI), Springer Nature Switzerland, 2024, pp. 749–760. https://doi.org/10.1007/978-3-031-72069-7_70.

[6] Balakrishnan, G., Zhao, A., Sabuncu, M., Guttag, J., Dalca, A., Voxelmorph: a learning framework for deformable medical image registration, IEEE Trans Med Imaging 38 (2019) 1788–1800. https://doi.org/10.1109/TMI.2019.2897538.

[7] Chlap, P., Finnegan, R. N., PlatiPy: processing library and analysis toolkit for medical imaging in Python, Journal of Open Source Software 8 (2023) 5374.

[8] Vaassen, F., Hazelaar, C., Vaniqui, A., Gooding, M., van der Heyden, B., Canters, R., van Elmpt, W., Evaluation of measures for assessing time-saving of automatic organ-at-risk segmentation in radiotherapy, Phys Imaging Radiat Oncol 13 (2020) 1–6. https://doi.org/10.1016/j.phro.2019.12.001.
